# A glimpse into a vanished ecosystem: reconstructing diet and palaeoenvironment of *Palaeoloxodon* from the Pleistocene of Taiwan

**DOI:** 10.1098/rsos.250935

**Published:** 2025-11-05

**Authors:** Deep Shubhra Biswas, Yogaraj Banerjee, David Baker, Chun-Hsiang Chang, Cheng-Hsiu Tsai

**Affiliations:** ^1^ Institute of Ecology and Evolutionary Biology, National Taiwan University, Taipei City 10617, Taiwan; ^2^ STARLAB, Department of Earth and Atmospheric Sciences, Central Michigan University, Mount Pleasant, MI 48859, USA; ^3^ School of Biological Sciences, Faculty of Science, The University of Hong Kong, Hong Kong; ^4^ Palaeontology Division, Geology Department, National Museum of Natural Science, Taichung 404023, Taiwan; ^5^ Department of Life Science, Tunghai University, Taichung 40704, Taiwan; ^6^ Department of Life Science, National Taiwan University, Taipei City 10617, Taiwan

**Keywords:** stable isotope, C_4_ diet, palaeohabitat, weaning, proboscidea

## Abstract

*Palaeoloxodon* is the largest known terrestrial mammal in the history of Taiwan. However, little is known about the palaeoecology and palaeoenvironment of these extinct proboscideans. In this study, we investigate the stable carbon and oxygen isotopic ratios of sequential enamel samples taken along the growth direction of molars from three *Palaeoloxodon* specimens of different ontogenetic ages from the Pleistocene of Taiwan. Our results suggest that these proboscideans relied extensively on a C_4_ diet, a dietary niche distinct from the Eurasian *Palaeoloxodon antiquus* but similar to the predominantly C_4_-grazing species *Palaeoloxodon namadicus*. The specimens show depleted *δ*^18^O values, indicating obligate drinking and reliance on running water sources, such as monsoon-fed rivers, which exhibit more depleted *δ*^18^O values. Additionally, the juvenile specimen shows an isotopic shift in both *δ*^13^C and *δ*^18^O values, aligning with the known signals of weaning in proboscideans. This could provide a novel insight into the life history and weaning age of *Palaeoloxodon*. Our results highlight, to our knowledge, the first evidence of a unique palaeohabitat of *Palaeoloxodon* from the Pleistocene of Taiwan, represented by C_4_ vegetation and the presence of a palaeo-river system that supported these giant proboscideans.

## 1. Introduction

The island of Taiwan is characterized by its intermittent connections with the Eurasian landmass owing to the glacial/interglacial cycles, and the faunal composition during the Pleistocene was markedly different from the Present, harbouring megafaunal clades that included the giant proboscidean *Palaeoloxodon* [1]. *Palaeoloxodon* was one of the most widespread proboscidean lineages in Afro-Eurasia. Studies indicate that these behemoths could reach extreme body sizes [2], with *Palaeoloxodon* from Taiwan estimated to have exceeded 10 tonnes in body mass [1]. Traditionally associated with vast woodland and grassland habitats, *Palaeoloxodon* exhibited a predominantly mixed-feeding strategy, with species from Africa, Europe and India favouring both browsing and grazing on C_3_ and C_4_ plants [3–12]. However, owing to the lack of similar studies on the Pleistocene fauna of Taiwan, the environment and ecosystem in which these megaherbivores thrived remain largely unknown.

As megaherbivores, proboscideans play a crucial role in their habitat as ‘ecosystem engineers’ [13,14]. They serve as valuable taxa for reconstructing past environments using a stable isotope approach, given their biology and land use patterns [11,15–18]. Stable isotope analysis has been an important tool for studying the ecology and biology of both extant and extinct mammals, with bulk samples of bioapatite providing a time-averaged record and serial sampling offering a time-series representation of an individual’s dietary history and environment [19,20]. In particular, dental enamel is an ideal material for isotope analysis, as it is the hardest biological substance and highly resistant to diagenetic alterations [21]. The carbon isotope composition of herbivore tooth enamel carbonate serves as an effective proxy of their diet [22–24], reflecting the differences in the photosynthetic pathways of C_3_ and C_4_ plants, which produce distinct *δ*^13^C ranges [25,26]. Similarly, the oxygen isotope value of bioapatite in mammalian tooth enamel corresponds to the *δ*^18^O of body water, which is influenced by oxygen uptake and loss during tooth development [27,28]. As herbivores that are evaporation insensitive, proboscideans reflect the stable oxygen isotope composition of their drinking water [29]. Thus, the *δ*^18^O value of their tooth enamel represents that of the available water source in the environment. In addition, proboscidean molars grow sequentially in succession through horizontal displacement during ontogeny [30,31], allowing for accurate ageing of individuals and providing insights into ontogenetic variation in isotopic signals, nursing periods and life history.

In this study, we performed stable isotope analysis of structural carbonate on serial enamel samples from three mandibles of Pleistocene *Palaeoloxodon*, representing different ontogenetic stages, recovered from the Taiwan Strait, to reconstruct their palaeoecology and palaeohabitat. We analysed *δ*^13^C to infer the diet of these proboscideans and *δ*^18^O as a proxy for the water sources they used for drinking. Furthermore, we compared published isotope data from bulk and serial samples of extant and extinct proboscideans to enhance understanding of the ecological differences among the *Palaeoloxodon* specimens from Taiwan and other species. We also discussed key aspects of the biology of these proboscideans, such as weaning age. Our results provide the first glimpse into the palaeoenvironment inhabited by *Palaeoloxodon* and associated fauna during the Pleistocene of Taiwan, at the easternmost boundary of continental Eurasia.

## 2. Material and methods

### 2.1. Locality and geological background

The sea bottom of the Taiwan Strait harbours an assemblage of vertebrate fossils, frequently collected during trawling activities by commercial fisheries. These trawling operations are carried out over a vast area at varying depths, leading to unclear reporting of the actual site and depth of collection. Alongside *Palaeoloxodon*, the locality has yielded fossils from the genera *Equus*, *Bubalus* and *Elaphurus* [32], *Panthera* and *Ursus* [33], *Crocuta* [34], *Alligator* [35], *Nyctereutes* [36], *Eschrichtius* [37], *Eubalaena* [38], *Mauremys* [39] and even a male Denisovan [40]. The nature of the assemblage has made it difficult to ascertain the exact geological age of the fossil site owing to the absence of associated sediments or microfossils. Tsai & Chang [38] proposed a broad time frame of the Middle to Late Pleistocene (0.78-0.01 Ma), which has since been widely adopted [1,39]. As for the terrestrial species, this geological age may be narrowed down to 70 000–10 000 years ago or 190 000–130 000 years ago [40] when the Taiwan Strait was land.

### 2.2. Studied materials

**Figure 1.**
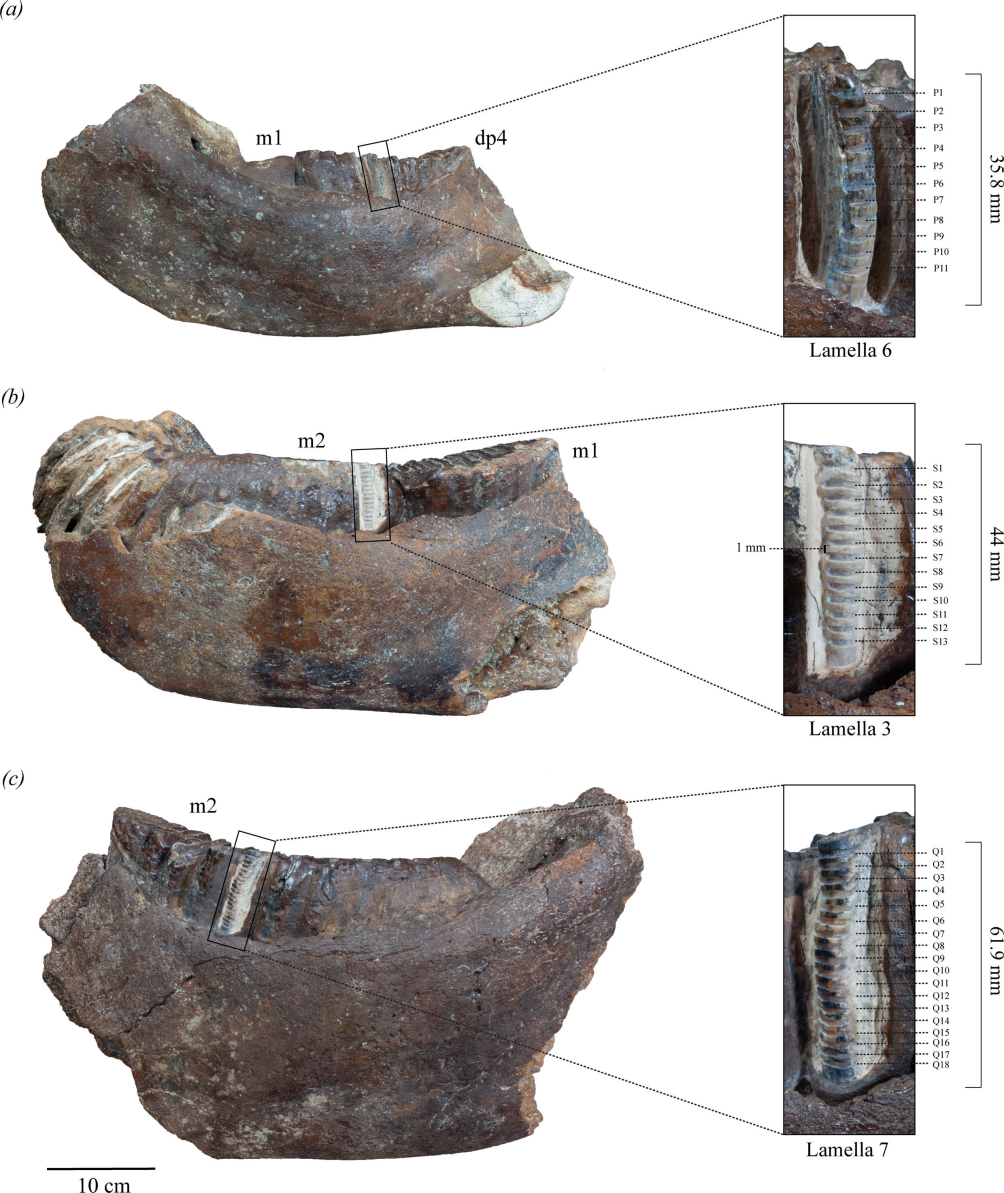
Studied mandibles of *Palaeoloxodon* from the Taiwan Strait and the serial sampling sites: (*a*) NTUM-VP 210127, (*b*) NTUM-VP 230921, and (*c*) NTUM-VP 230920.

A total of three mandibles of *Palaeoloxodon* are included in our study: NTUM-VP 210127, NTUM-VP 230920 and NTUM-VP 230921 (figure 1). These specimens are under the permanent curation of the laboratory of evolution and diversity of fossil vertebrates, Museum of Zoology, National Taiwan University (NTU), Taipei, Taiwan. A lower m3 specimen of extant *Elephas maximus*, N-000033/NF002445, from the collection of National Museum of Natural Science (NMNS) in Taichung, Taiwan, was also sampled in our study for comparison. Furthermore, published stable carbon and oxygen isotope data from bulk and serial samples of proboscidean species from the Pliocene to the present were compiled for comparison with our results (electronic supplementary material, table S1; [3,4,6,8–11,41]). Given the scope of this study, we avoided the taxonomic complexities at subspecies levels within the *Palaeoloxodon recki* and *Loxodonta adaurora* complexes, and grouped all subspecies-level taxa under *P. recki sensu lato* (s. l.) and *L. adaurora sensu lato* (s. l.), respectively.

The ontogenetic ages of the studied *Palaeoloxodon* specimens were estimated following the criteria proposed by Takahashi & Chang [42] for *Palaeoloxodon naumanni*. Although the ageing method by Laws [30] for *Loxodonta africana* is widely used for extinct proboscidean species, significant differences exist in the molar growth progression, wear stages and morphology between *Loxodonta* and *Palaeoloxodon* [42,43]. NTUM-VP 210127 is a left mandible that preserves a dp4 and m1, with the dp4 in use and the m1 showing no occlusion. Based on the ageing criteria of Takahashi & Chang [42], the mandible belonged to a juvenile individual with an estimated age of 5 years at the time of death. NTUM-VP 230921 is a left mandible with an m1 in advanced wear and an m2 in early wear and is attributed to a subadult individual with an approximate age of 15.5 years at the time of death. NTUM-VP 230920 is a right mandible with an m2 in the mid-wear, with all plates in wear, indicating an estimated age range of 24–27 years at the time of death. Three-dimensional models of the three sampled *Palaeoloxodon* are associated with the article online and are freely available (electronic supplementary material, S2, section 1).

### 2.3. Sample collection and pre-treatment

The best preserved molar plates in occlusion with the longest crown lengths were selected for serial sampling (figure 1). This process was aided by computed tomography (CT) scans to avoid sampling plates with damaged enamel surfaces. The cementum was manually cleaned from the selected molar plates using an airscribe tool and a diamond-tipped rotary drill to avoid contamination before collecting enamel samples. CT scans indicated that the enamel thickness varied apicocervically along a given lamella. Previous studies have suggested that inner enamel provides stronger isotopic signals and more accurately represents changes over time, with reduced damping [9,44–46]. Damping arises during enamel growth, when the original isotopic signal gets time averaged as mineralization and maturation progress. To minimize this effect, approximately 1 mm of the outer enamel layer was removed, taking into account the relatively thin enamel of the specimens (mean measured enamel thickness at occlusal surface = 1.53 mm), and following established protocols for proboscideans [9,46].

The enamel growth rate for proboscideans is known to range between 8 and 21 mm yr^−1^, showing interspecific variations [47–51]. Dirks *et al.* [48] reported a slower plate extension rate in the dwarf species *Palaeoloxodon cypriotes* (enamel growth rate of approx. 8.42 mm yr^−1^) compared to the mainland *Mammuthus columbi*, with both species exhibiting variability in extension rates from the apex to the cervix of molar lamella. The variation in enamel thickness observed in the studied *Palaeoloxodon* specimens from Taiwan may be owing to such differences in extension rate. The study further suggested that the higher extension rate could be attributed to the relatively higher crown height of *M. columbi*. However, because of limited research on mainland *Palaeoloxodon* species, it remains unclear whether they shared similar enamel extension rates with dwarf species or exhibited faster growth rates like mainland *Mammuthus* species. Thus, for the scope of our study, serial samples were collected at 1 mm intervals to approximate a monthly resolution of stable isotope data from the specimens.

Powdered enamel samples were collected using a diamond-tipped rotary drill, creating parallel 1–2 mm grooves perpendicular to the growth axis, extending from the occlusal end to the base of the molar crown on the lingual side of the specimens (figure 1). The sampling surface and utensils were cleaned with an air compressor blower and 75% ethyl alcohol after each groove was sampled to prevent cross-contamination. Furthermore, samples were collected from selected grooves of each specimen to check for diagenesis using X-ray diffraction (XRD; electronic supplementary material, S2, section 2, figure 1). Although recent research on fossil proboscidean molars has shown that diagenesis does not affect enamel characteristics [21], testing the integrity of enamel physical properties using XRD was necessary given that the studied specimens were preserved in saline conditions.

To remove diagenetic carbonates and organic matter, the enamel samples were first treated with 40% commercial bleach solution (approx. 2.5% NaOCl) for 18–24 h at 25°C [9]. The Eppendorf tubes were then centrifuged 4–5 times at 3200 rpm for 4 min until the samples were separated from the supernatant [41]. Following this, the supernatant was removed, replaced with distilled water, and centrifuged again. The process was repeated three times to neutralize the pH. Subsequently, 0.1 ml of 0.1 M acetic acid per 1 mg of enamel was added to each sample using separate pipettes [52]. The tubes were agitated for proper mixing and then left to sit for no more than 10 min, as extended acid treatment can lead to sample dissolution and altered isotopic signals [52,53]. They were then centrifuged for 4 min at 3200 rpm, followed by replacing the acetic acid with distilled water using a clean pipette. The tubes were centrifuged again for another 4 min, and the washing process was repeated 3–4 times until the acid was neutralized. Finally, the tubes were placed in a hot-air oven set at 60°C to dry the samples for 24 h.

### 2.4. Stable carbon and oxygen isotope of enamel carbonates

The stable isotope analysis was conducted at the Stable Isotope Laboratory, School of Biological Sciences, The University of Hong Kong, Hong Kong. Approximately 1–1.5 mg of pre-treated enamel samples were weighed using a Mettler Toledo XPR6UD5 microbalance for analysis. The samples were then reacted with phosphoric acid using an Elementar Isoflow headspace analyser set at a tray temperature of 70°C. The released CO_2_ from the reaction was analysed using an Elementar Isoprime Precision isotope-ratio mass spectrometer to determine the *δ*^13^C and *δ*^18^O values of the samples. The analysis included replicates of reference standards NBS 18 and IAEA 603 to ensure precision.

The stable carbon and oxygen isotope ratios are reported in delta (*δ*) notation relative to the Vienna-Pee Dee Belemnite (VPDB) standard, where:


$$\delta_{\text{sample }}\;‰=\left(R_{\text{Sample }}/R_{\text{Standard }}-1\right)\times 1000.$$

Here, *R*_Sample_ denotes the ratio of ^13^C/^12^C and ^18^O/^16^O of the sample, and *R*_Standard_ refers to the corresponding ratios of the VPDB standard. For the published datasets where *δ*^18^O values were reported relative to Vienna Standard Mean Ocean Water (VSMOW) standards, conversion to VPDB standard was done using the following equation [17]:


$$\delta^{18}O(‰VPDB)=\left\{\delta^{18}O(‰\text{ VSMOW })-30.91\right\}/1.03091.$$

Cerling & Harris [24] reported that the *δ*^13^C value of the diet of large extant mammals, including elephants, is depleted by 14.1‰ relative to enamel because of metabolic fractionation. This enrichment factor is necessary when inferring diet from enamel samples. Additionally, variations in the isotopic composition of the atmosphere through the geological times must also be considered, as it influences the *δ*^13^C values of terrestrial plants. The *δ*^13^C_atmCO2_ is estimated at −6.3‰ during the Pliocene, −6.5‰ during the Pleistocene and −8‰ following the Industrial Revolution [3,54–56]. Thus, the *δ*^13^C_enamel_ results were converted to *δ*^13^C values corresponding to the modern equivalent diet (*δ*^13^C_diet-meq_) for the studied specimens and published data by accounting for the diet-enamel enrichment factor and the difference in *δ*^13^C_atmCO2_.

For the interpretation of *δ*^13^C results and comparison with other taxa, the following vegetation classification was used: (i) closed-canopy forest, −20.5 to −14.5‰; (ii) woodland-mesic C_3_ grassland, −14.5 to −9.5‰; (iii) open woodland-xeric C_3_ grassland, −9.5 to −6.5‰; (iv) mixed C_3_–C_4_ grassland, −6.5‰ to −1.5‰; and (v) pure C_4_ grassland, −1.5‰ to +6.5‰ [17,57]. These categories were further modified to represent the *δ*^13^C_diet-meq_ values by incorporating the enrichment factor of −14.1‰. Although these criteria suggest clear limits between vegetation types, the isotopic signals should be interpreted as reflecting contributions from multiple vegetation sources. Thus, to further quantify the proportional abundance of C_4_ vegetation in the diet of the studied specimens, we applied a two-member mixing model using −7.6‰ and −34.6‰ as the C_4_ and C_3_ end members, respectively. We used the Wilcoxon rank-sum test (with Holm adjustment), a non-parametric method considering the non-normal distribution and limited sample size for some taxa, to compare the *δ*^13^C_diet-meq_ and *δ*^18^O values of the *Palaeoloxodon* specimens from Taiwan with those of other extinct and extant proboscidean taxa. We calculated the effect sizes using rank-biserial correlation to further aid the statistical comparison. As this comparison included published data from both bulk and serial enamel samples, the mean *δ*^13^C_diet-meq_ and *δ*^18^O values of serial samples were used to ensure consistency with the time-averaged data represented by bulk samples.

## 3. Results

### 3.1. Stable carbon and oxygen isotope of enamel carbonate

**Figure 2.**
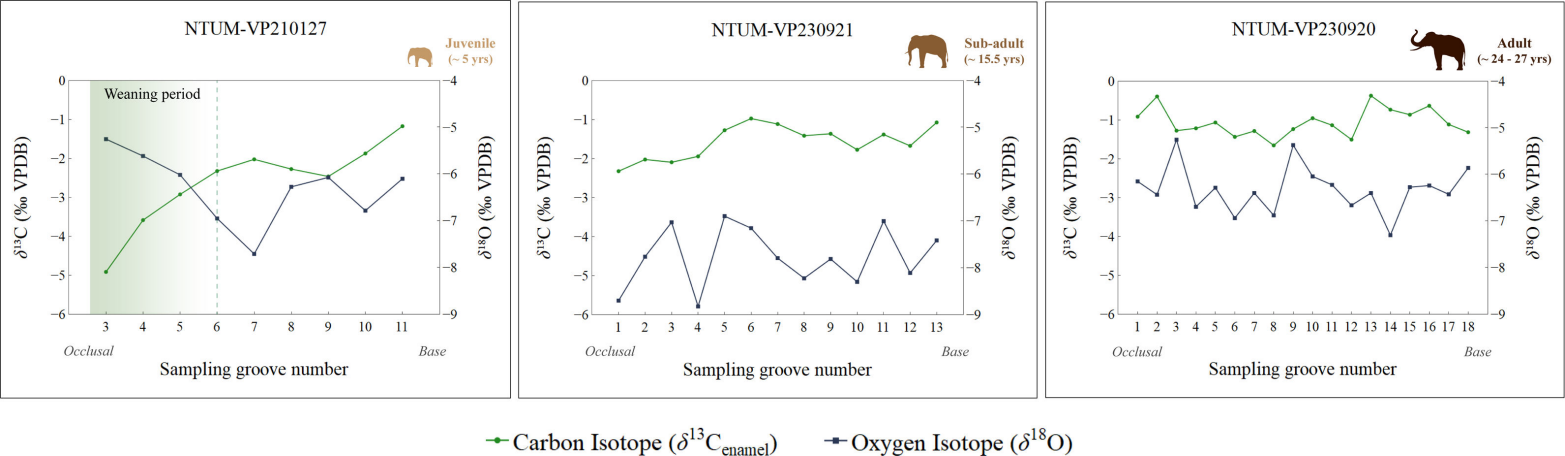
Stable carbon (*δ*^13^C_diet-meq_) and oxygen (*δ*^18^O) isotope values of the studied *Palaeoloxodon* specimens. The plots were prepared using the ‘plotly’ package [58] on R version 4.3.2 [59]. Illustrations produced by DSB.

The *δ*^13^C_enamel_ values of the studied *Palaeoloxodon* specimens range from −4.9‰ to −0.4‰ (*δ*^13^C_diet-meq_ ranging from −17.5‰ to −13‰), indicative of a predominantly C_4_ diet, with a mean value of −1.6‰ (mean *δ*^13^C_diet-meq_ = −14.2‰; standard deviation, *σ* = 0.85‰; table 1). The isotope values of the subadult and adult specimens, NTUM-VP 230921 and NTUM-VP 230920, respectively, show a narrower range of variation between −2.3‰ and −0.4‰ (*δ*^13^C_diet-meq_ = −14.9‰ and −13‰). NTUM-VP 230921 has *δ*^13^C_enamel_ values ranging from −2.3‰ to −1.0‰ with a mean value of −1.6 ‰ (*δ*^13^C_diet-meq_ = −14.9‰ and −13.6‰; mean *δ*^13^C_diet-meq_ = −14.2‰; *σ* = 0.43‰), while for NTUM-VP 230920 it ranges from −1.7‰ to −0.4‰ with a mean of −1.1‰ (*δ*^13^C_diet-meq_ = −14.3‰ to −13‰; mean *δ*^13^C_diet-meq_ = −13.7‰; *σ* = 0.36‰). Conversely, the juvenile specimen, NTUM-VP 210127, exhibits a highly negative value of −4.9‰ (*δ*^13^C_diet-meq_ = −17.5‰) at sampling groove 3 (near the occlusal surface), which gradually increases before eventually falling within the range of the larger specimens at sampling groove 6. It has a mean value of −2.6‰ (mean *δ*^13^C_diet-meq_ = −15.2‰; *σ* = 1.09‰; figure 2). Sampling grooves 1 and 2 for this specimen did not yield results owing to insufficient enamel powder for analysis. The two-member mixing model estimations indicate that NTUM-VP 210127 had a C_4_ dietary contribution ranging from 63.3% to 77.1%, NTUM-VP 230921 from 72.9% to 77.9%, and NTUM-VP 230920 from 75.3% to 80%, consistent with a C₄-dominated diet (electronic supplementary material, S2, table S1).

**Table 1.** Stable isotope results for serial samples from *Palaeoloxodon* and *Elephas maximus* (extant) specimens in this study.

specimen ID	taxa	age (years)	sample ID	*δ*^13^C_enamel_ (‰)	*δ*^13^C_diet-meq_ (‰)	*δ*^18^O (‰)
NTUM-VP 210127	*Palaeoloxodon* sp*.*	approx. 5	P1	_	_	_
			P2	_	_	_
			P3	−4.92	−17.52	−5.26
			P4	−3.59	−16.19	−5.62
			P5	−2.93	−15.53	−6.02
			P6	−2.33	−14.93	−6.96
			P7	−2.03	−14.63	−7.72
			P8	−2.28	−14.88	−6.28
			P9	−2.47	−15.07	−6.08
			P10	−1.88	−14.48	−6.79
			P11	−1.18	−13.78	−6.11
NTUM-VP 230921	*Palaeoloxodon* sp*.*	approx. 15.5	S1	−2.33	−14.93	−8.71
			S2	−2.03	−14.63	−7.77
			S3	−2.1	−14.7	−7.03
			S4	−1.95	−14.55	−8.83
			S5	−1.28	−13.88	−6.9
			S6	−0.98	−13.58	−7.16
			S7	−1.12	−13.72	−7.8
			S8	−1.42	−14.02	−8.23
			S9	−1.37	−13.97	−7.82
			S10	−1.78	−14.38	−8.3
			S11	−1.39	−13.99	−7.01
			S12	−1.68	−14.28	−8.11
			S13	−1.08	−13.68	−7.42
NTUM-VP 230920	*Palaeoloxodon* sp*.*	approx. 24–27	Q1	−0.92	−13.52	−6.16
			Q2	−0.4	−13	−6.44
			Q3	−1.28	−13.88	−5.26
			Q4	−1.22	−13.82	−6.7
			Q5	−1.07	−13.67	−6.29
			Q6	−1.44	−14.04	−6.94
			Q7	−1.29	−13.89	−6.4
			Q8	−1.66	−14.26	−6.88
			Q9	−1.24	−13.84	−5.38
			Q10	−0.96	−13.56	−6.05
			Q11	−1.14	−13.74	−6.23
			Q12	−1.51	−14.11	−6.67
			Q13	−0.38	−12.98	−6.4
			Q14	−0.74	−13.34	−7.31
			Q15	−0.87	−13.47	−6.28
			Q16	−0.64	−13.24	−6.24
			Q17	−1.12	−13.72	−6.43
			Q18	−1.32	−13.92	−5.87
N-000033/NF002445	*Elephas maximus*	NA	T1	−13.18	−27.28	−7.11
			T2	−13.62	−27.72	−6.78
			T3	−12.84	−26.94	−6.58
			T4	−13.07	−27.17	−6.75
			T5	−13.51	−27.61	−7.74
			T6	−12.9	−27	−6.52
			T7	−13.17	−27.27	−6.94
			T8	−12.77	−26.87	−8.05
			T9	−12.27	−26.37	−6.67
			T10	−12.88	−26.98	−6.11

The serial samples of these specimens show an overall negative value for *δ*^18^O, ranging between −8.8‰ and −5.3‰, with a mean of −6.8‰ (*σ* = 0.91‰; table 1), characteristic of obligate drinking in mammals. The adult specimen, NTUM-VP 230920, has a *δ*^18^O value ranging from −7.3‰ to −5.3‰ (mean = −6.3‰; *σ* = 0.5‰), while the subadult, NTUM-VP 230921, exhibits a comparatively higher fluctuation with values ranging from −8.8‰ to −6.9‰ (mean = −7.8‰; *σ* = 0.65‰). Furthermore, the juvenile specimen shows a similar trend for *δ*^18^O, mirroring the *δ*^13^C_diet-meq_ pattern, with values ranging from −7.7‰ to −5.3‰ (mean = −6.3‰; *σ* = 0.74‰). NTUM-VP 210127 has a more positive *δ*^18^O value of −5.3‰ at sampling groove 3, which then decreases almost linearly to −7.7‰ at sampling groove 7. It then rises and follows an undulating pattern similar to the other specimens (figure 2).

The studied *E. maximus* specimen N-000033/NF002445 exhibits *δ*^13^C_enamel_ values ranging from −13.6‰ to −12.3‰ (*δ*^13^C_diet-meq_ = −27.7‰ to −26.4‰; mean δ^13^C_diet-meq_ = −27.1‰; *σ* = 0.39‰; table 1; electronic supplementary material, S2), which is slightly more negative than the range reported in the literature for extant *E. maximus* (−11.9‰ to −7.2‰) [9]. The *δ*^18^O values for this specimen range from −8.1‰ to −6.1‰ (mean = −6.9‰; *σ* = 0.58‰), which fall within the range of the published values for extant *E. maximus* (−15.9‰ to −2.5‰) [9].

### 3.2. Comparison with other proboscidean taxa

We compared the *δ*^13^C_diet-meq_ and *δ*^18^O results of *Palaeoloxodon* from our study with other extinct and extant proboscidean taxa, including species within the genera *Loxodonta* [3], *Elephas* [9,41] and *Palaeoloxodon* [3,4,6,8–11]. The molar specimen of extant *E. maximus* (N-000033/NF002445), analysed in this study, serves as a modern reference and is included alongside the published data for extant *E. maximus* to support the comparison.

**Figure 3.**
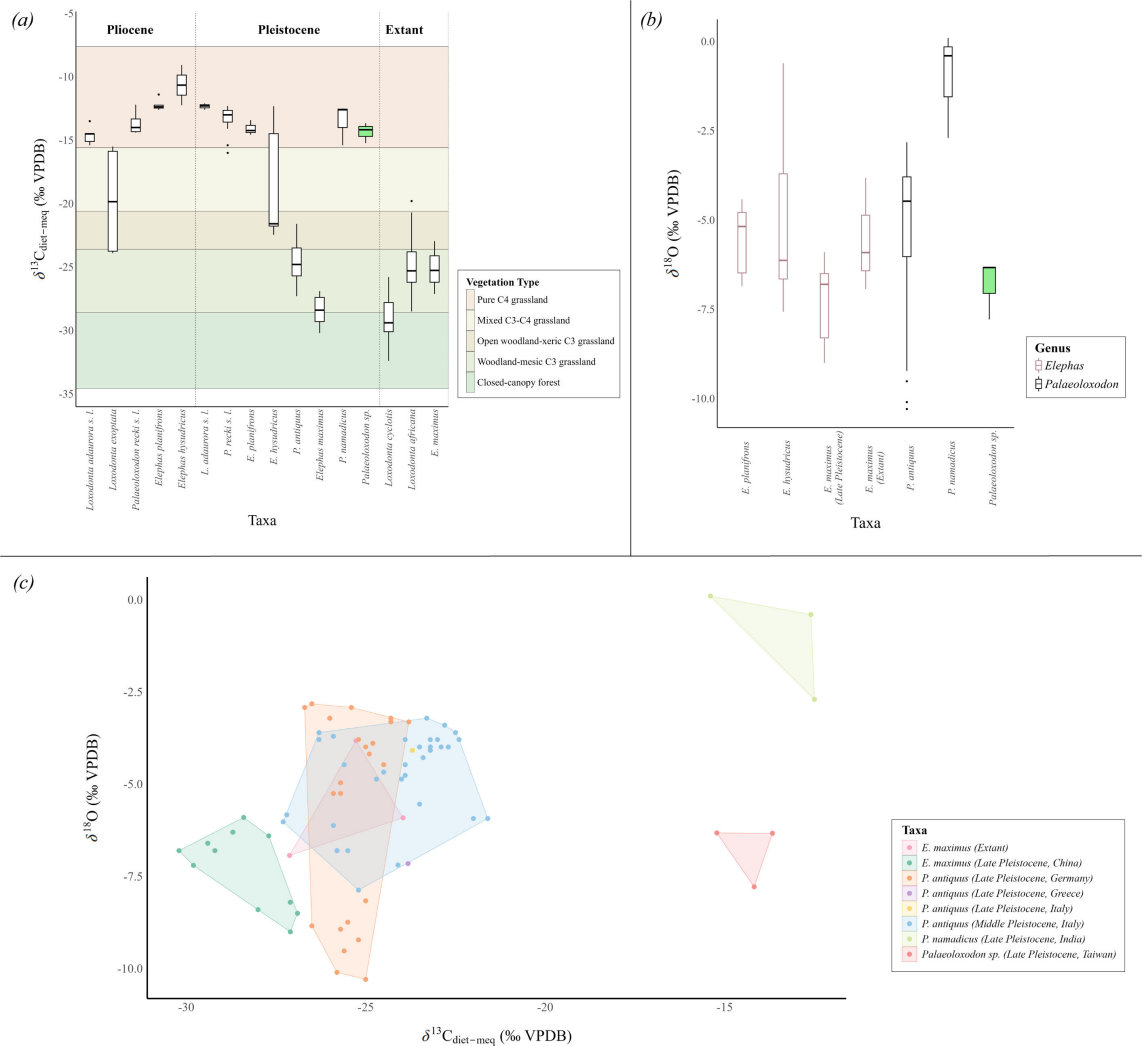
Comparison of serial and bulk stable carbon and oxygen data: (*a*) Box plots of *δ*^13^C_diet-meq_ values for extinct and extant species of *Loxodonta* [3], *Elephas* ([9,41]; this study), and *Palaeoloxodon* ([3,4,6,8–11]; this study); (*b*) box plot of *δ*^18^O values for extinct and extant *Elephas* and *Palaeoloxodon* species; and (*c*) scatter plot of *δ*^18^O versus *δ*^13^C_diet-meq_, comparing isotope data for species within the genera *Elephas* and *Palaeoloxodon*. The green box plot represents the serial samples for *Palaeoloxodon* from Taiwan. All the plots are prepared on R, version 4.3.2 [59] using the ‘ggplot2’ package [60].

The *δ*^13^C_diet-meq_ values of all the compared proboscidean taxa vary over a wide range, from −32.4‰ to −9.3‰. The values for the studied *Palaeoloxodon* specimens align with some Pliocene and Pleistocene members from the genera *Elephas* and *Loxodonta* but are markedly different from extant species (figure 3*a*). In the genus *Palaeoloxodon*, the *δ*^13^C_diet-meq_ values for *Palaeoloxodon* from Taiwan (−15.2‰ to −13.7‰) fall within the range of *P. namadicus* specimens (−15.4‰ to −12.5‰) while being distinctly different from *P. antiquus* (−27.3‰ to −21.6‰). A significant difference in diet is also observed between *Palaeoloxodon* from Taiwan and *L. africana* (*W* = 147, *p* = 0.043), *Loxodonta cyclotis* (*W* = 87, *p* = 0.049) and *P. antiquus* (*W* = 171, *p* = 0.043) based on the Wilcoxon rank sum test (electronic supplementary material, S2, table S2). Furthermore, the rank-biserial correlation analysis shows a strong positive effect (*r* = 1) for *Loxodonta exoptota*, *P. antiquus*, late Pleistocene *E. maximus*, extant *E. maximus*, *L. cyclotis* and *L. africana*, indicating that these taxa exhibit significantly more negative *δ*^13^C_diet-meq_ values than *Palaeoloxodon* from Taiwan (electronic supplementary material, S2, table S3). Additionally, a moderate negative effect (*r* = −0.67) is observed for *Elephas planifrons*, suggesting more enriched *δ*^13^C_diet-meq_ values when compared with *Palaeoloxodon* from Taiwan. Conversely, weak to moderate effects are seen for *L. adaurora*, *P. recki*, *Elephas hysudricus* and *P. namadicus*, but since the confidence intervals (CI = 0.95) include zero, these trends are considered uncertain.

The *δ*^18^O values for all the proboscidean taxa show a range, from −10.3‰ to 0.1‰. In contrast to the *δ*^13^C_diet-meq_ values, *δ*^18^O values for *Palaeoloxodon* from Taiwan (−7.8‰ to −6.3‰) are markedly different from the range observed in *P. namadicus* (−2.7‰ to 0.1‰) but closely align with *P. antiquus* and other species within the genus *Elephas* (figure 3*b*,*c*). The Wilcoxon rank sum test did not detect any significant difference between the compared taxa (electronic supplementary material, S2, table S2). However, the rank-biserial correlation suggests strong negative effects for *P. namadicus* (*r* = −1), while *P. antiquus* shows a moderate negative effect (*r* = −0.59), indicating significantly higher *δ*^18^O values in these taxa compared to *Palaeoloxodon* from Taiwan (electronic supplementary material, S2, table S3). Additionally, moderate to weak negative effects are also observed for *E. planifrons*, *E. hysudricus* and extant *E. maximus*, while a weak positive effect is observed for late Pleistocene *E. maximus*. However, these effects are deemed uncertain because of the confidence intervals (CI = 0.95) including zero.

## 4. Discussion

### 4.1. Dietary niche and palaeohabitat of *Palaeoloxodon* from Taiwan

The *δ*^13^C values recorded for the *Palaeoloxodon* specimens from the Taiwan Strait provide new insights into the ecosystem that persisted in Taiwan during the Pleistocene. All studied *Palaeoloxodon* specimens, representing different age groups, showed extensive reliance on C_4_ vegetation as part of their diet. Furthermore, our results for the subadult (NTUM-VP 230921) and adult (NTUM-VP 230920) individuals illustrate that they fed on C_4_ plants almost throughout the year, suggesting the constant availability in their habitat. This is indicative of a warm, predominantly arid environment with physical conditions that favour C_4_ over C_3_ vegetation, without major seasonal shifts in plant community structure. Plants using the C_4_ pathway possess a metabolic advantage, allowing them to proliferate and outcompete C_3_ vegetation in dry, highly saline conditions with low CO_2_ and high temperatures [61–63]. Consequently, C_4_ plants, particularly grass species, dominate modern warmer ecosystems such as savannahs at lower latitudes, while C_3_ species are more abundant in higher latitude regions [62,64]. The evolution of C_4_ vegetation was aided by a decrease in atmospheric CO_2_ levels during the Oligocene, with further expansion into lower latitudes during the late Miocene and subsequent glaciation events of the Pleistocene [63,65–69]. Similar expansion and prevalence of C_4_-dominated grasslands have been reported from eastern Asia during the Pleistocene into the Holocene, with an increase in C_4_ abundance at lower latitudes [70–75]. Thus, our findings align with the global distribution pattern observed for C_4_ plants during this period. The co-occurrence of other grassland-associated taxa, such as *Equus*, *Elaphurus*, *Bubalus* and *Crocuta*, in the fossil record of the Taiwan Strait further supports the presence of grassland habitats [32,34]. In contrast to the C_4_-dominated habitat from the Pleistocene, present-day Taiwan is represented by a different range of ecological zones, from subtropical high-mountain coniferous woodlands and montane cloud forests to tropical evergreen and deciduous broad-leaved forests [76,77]. Our results thus suggest that the Pleistocene environment of the Taiwan Strait was ecologically distinct, providing a unique habitat in which megaherbivores like *Palaeoloxodon* thrived. However, consequent studies on stable isotope signatures for other fossil taxa from the assemblage are necessary for a holistic understanding of this vanished ecosystem in the eastern margin of continental Eurasia.

**Figure 4.**
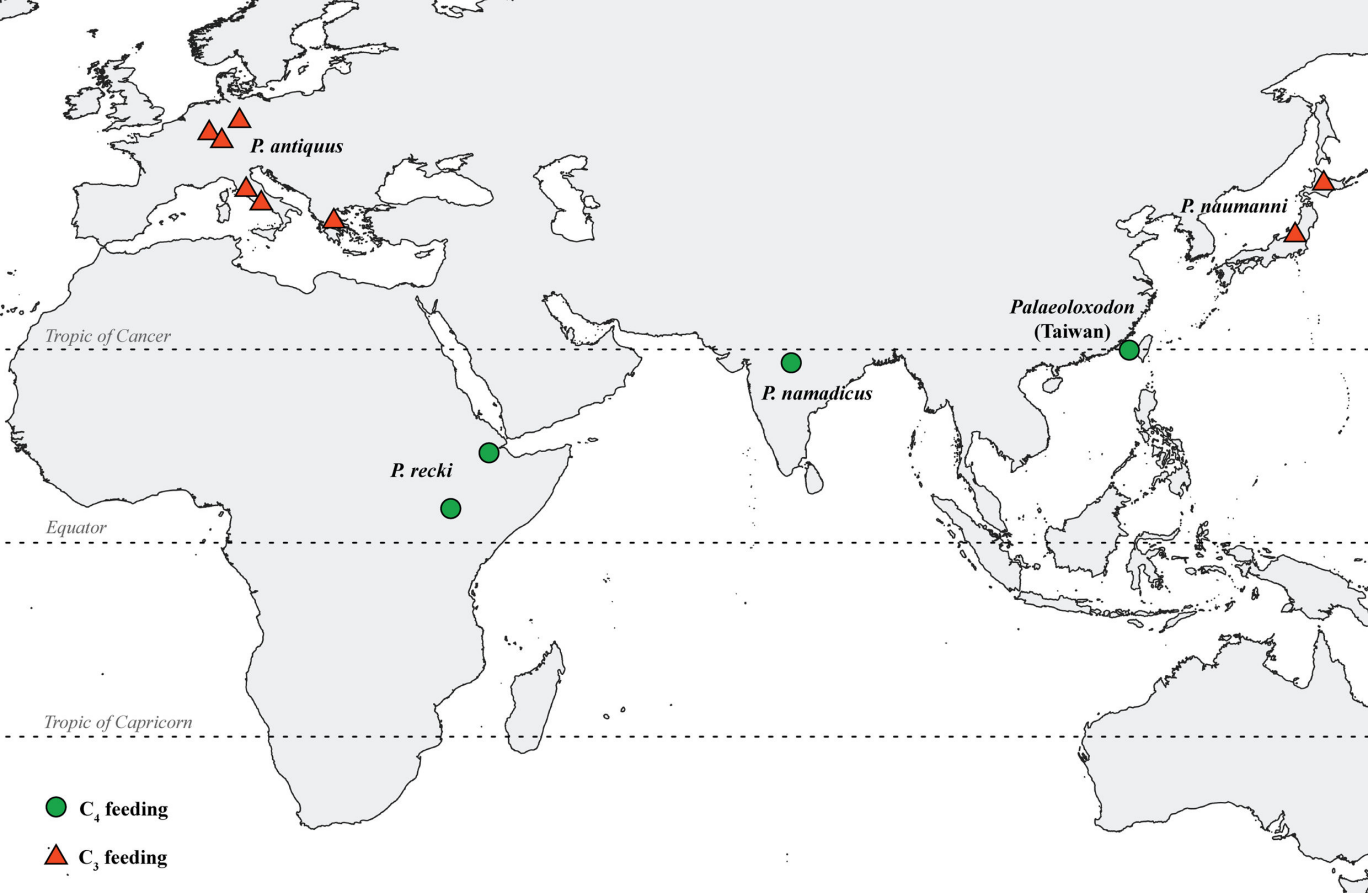
Map showing dietary preferences of different *Palaeoloxodon* species.

**Figure 5.**
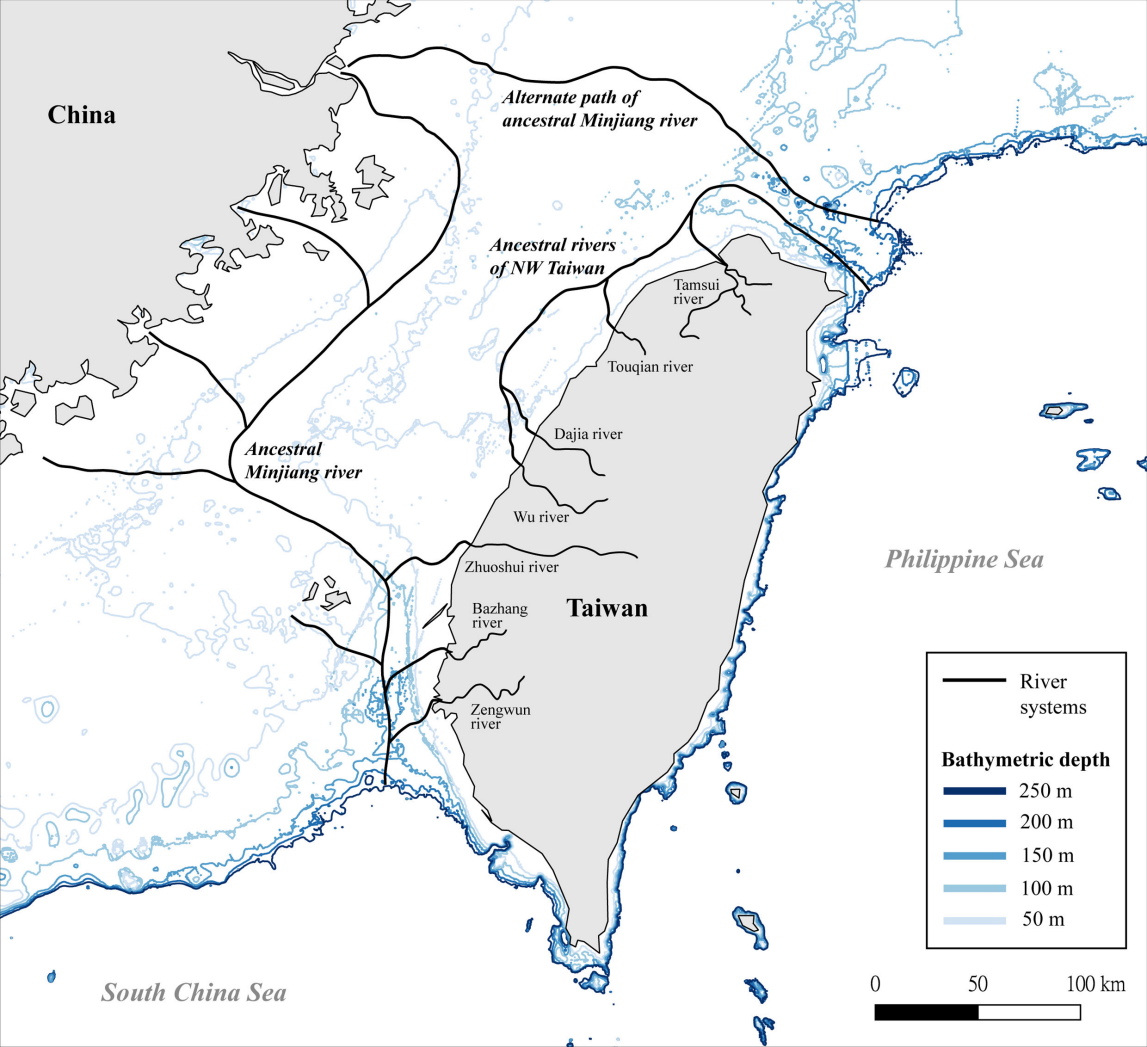
Map depicting the postulated palaeo-river system on the Taiwan continental shelf during the Pleistocene, alongside present-day major rivers and bathymetric depth data. Modified from Boggs *et al*. [106]. The map was created in QGIS using bathymetric data from GEBCO (https://www.gebco.net/).

We further explored the spatial heterogeneity in the dietary pattern of *Palaeoloxodon*. The dietary niche of *Palaeoloxodon* from Taiwan is markedly different from extant proboscidean species, which are predominantly C_3_ herbivores ([3,9]; this study). Within the genus *Palaeoloxodon*, the diet differs significantly from that of *P. antiquus* from Europe ([4,6,8,10,11]; figure 3) and the Japanese insular species *P. naumanni*, as recently reported by Naito [78], both of which relied on a C_3_ diet. By contrast, *P. recki* from the Plio-Pleistocene of Africa and *P. namadicus* from the Late Pleistocene of India exhibited a predominantly C_4_ diet, indicating grazing preferences [3,9] similar to those of *Palaeoloxodon* from Taiwan (figure 3). Similarly, studies using mesowear angle analysis have suggested a strong grazing signal in *P. recki* specimens [79,80]. A recent study on serial enamel samples of *P. recki* and *Palaeoloxodon jolensis* from the late Early and Middle Pleistocene formations of Ethiopia, Africa, further confirmed a predominantly C_4_ diet for these archaic *Palaeoloxodon* species [81]. This pattern agrees with the broader dietary shifts observed in multiple proboscidean lineages from the late Miocene to Pleistocene, when reliance on C_4_-dominated grasslands increased in the tropical and subtropical regions following the global C_4_ expansion [3,46]. Further support to this transition is seen in specimens of *L. adaurora* and *E. planifrons* from the Pliocene and Pleistocene from Africa and India, which were also C_4_ grazers ([3,9]; figure 3). Similarly, *E. hysudricus* was a C_4_ grazer during the Early Pleistocene of India before being replaced by *P. namadicus* in the grazing niche during the Middle Pleistocene [9]. These observations underscore the ecological adaptability of crown elephantids to grassland habitats in the tropics and subtropics, with the *Palaeoloxodon* lineage showing particularly high grazing adaptations. Our findings further add to this understanding that *Palaeoloxodon* from Taiwan represents a subtropical species that relied near-exclusively on C_4_ plants, similar to *P. recki* and *P. namadicus*, while *P. antiquus* thrived in mixed habitats at higher latitudes where C_3_ vegetation remained dominant (see figure 4; [4,5,8]).

The presence of such megaherbivore clades in a habitat that has no remnants in present-day Taiwan highlights the importance of comprehensive research on these now-lost ecosystems to understand the faunal turnover that gave rise to the present biodiversity. Further investigations looking into the geochemical, biochronological and trophic network aspects of these assemblages, such as food web structure and community connectedness, could enhance our understanding of the extinction event that occurred in the southeastern margin of Eurasia and its underlying effects. Moreover, given the significant role played by C_4_ plants in global carbon assimilation [82], it is crucial to better understand vanished C_4_-dominated ecosystems and the underlying causes of their disappearance. Thus, future research should also focus on applying a multi-proxy approach that integrates mesowear and microwear techniques with stable isotope data to study these proboscideans and the associated fauna from the Taiwan Strait.

### 4.2. Stable isotope-based insights into the palaeobiology and life history of *Palaeoloxodon*

Our study provides crucial insights into the life history and biology of *Palaeoloxodon* from Taiwan. Although our stable isotope results suggest the persistence of a relatively stable environment, the comparatively higher standard deviations of *δ*^13^C_diet-meq_ and *δ*^18^O values for the subadult specimen, NTUM-VP 230921 (*σ*_*δ*13C_ = 0.43‰, *σ*_*δ*18O_ = 0.65‰), in comparison to the adult (*σ*_*δ*13C_ = 0.36‰, *σ*_*δ*18O_ = 0.5‰), can be indicative of a broader landscape use in the former (figure 2). In extant elephants, herds display extensive land use patterns in family groups that include subadult individuals. Such movements are driven by the availability of foraging sites and water across the landscape [15,83,84]. Furthermore, subadult male elephants leave the protection of the natal herd between the ages of 9–18 years [85], following which they move in a broader range, often as part of male-only groups [86–88]. Similar patterns have also been observed in non-elephantid proboscideans like *Mammut americanum* that display increased land use patterns in subadult bull mastodons after separation from the natal herd [89]. Thus, the minimal variation observed in our results for NTUM-VP 230921 aligns with the movement behaviours seen in subadult proboscideans, both as part of their natal herds and post-separation. This could be indicative of social and behavioural patterns in these extinct proboscideans similar to those of extant elephant species. Alternatively, such variations could also be attributed to damping effects caused by irregularity in sampling depths or enamel thickness along the sampled area, which cannot be ruled out.

In addition, the stable isotope data for the juvenile specimen (NTUM-VP 210127) exhibit patterns consistent with weaning behaviour. The observed values for *δ*^13^C_enamel_ and *δ*^18^O between sampling grooves (3–6 for *δ*^13^C_enamel_ and 3–7 for *δ*^18^O; table 1; figure 2) suggest a dietary transition from nursing on maternal milk to independent feeding on plant matter. While nursing and weaning in extinct proboscideans have been sparsely understood, studies have illustrated that such behaviour can be identified using stable isotope approaches [90,91]. The *δ*^13^C value of milk is generally lower than that of the dietary matter consumed by the mother, owing to the more depleted values of milk fat in comparison to the carbohydrates derived from foraging [92–95]. Considering that elephant milk has a higher content of fat in comparison to bovine and human milk [96], it is expected to be highly depleted in *δ*^13^C. Thus, in the case of the juvenile specimen in our study, the high negative values for *δ*^13^C_enamel_ at groove 3 (table 1), followed by progressive enrichment in subsequent sampling grooves, is consistent with a gradual decrease in milk consumption and increased incorporation of solid food in the diet. This interpretation is consistent with the findings of Metcalfe *et al.* [91], who reported that nursing in *Mammuthus primigenius* is associated with a decreased *δ*^13^C value of enamel samples (approx. 1.5‰ more negative). Similarly, the gradual decrease in the *δ*^18^O value from grooves 3 to 7 further aligns with the pattern observed in *δ*^13^C_enamel_, given that water in milk is typically more enriched than drinking water [95,97–101]. Higher *δ*^18^O values of tooth enamel from juvenile proboscideans have also been reported in other studies [91,101]. The pattern observed in the *δ*^18^O value thus further corroborates the δ^13^C_enamel_ variation as indicative of weaning in the juvenile *Palaeoloxodon* specimen. Thus, our results highlight that juveniles of *Palaeoloxodon* included a fair amount of maternal milk in their diet until the age of approximately 5–6 years, similar to that of extant proboscideans [102,103], before completely transitioning to solid food. Further incorporation of *δ*^15^N into the analysis could provide added evidence of nursing and weaning, as *δ*^15^N is a better proxy of these processes in proboscideans, as illustrated by earlier studies [90,91,101].

### 4.3. Palaeo-river system of the Taiwan Strait

The presence of a palaeo-river system in the Taiwan Strait is debated owing to lack of dedicated research. However, the occurrence of freshwater taxa such as *Alligator* [35] and *Mauremys* [39] in this fossil assemblage, suggests the presence of freshwater habitats in this region during the Pleistocene. The stable oxygen isotope results for the *Palaeoloxodon* specimens from our study provide further understanding about the palaeohydrology of the region during the Pleistocene. Despite the similarity in their dietary niche, these specimens differ markedly from *P. namadicus* based on the observed *δ*^18^O values, which show a highly depleted range (figure 3*b*,*c*). Patnaik *et al.* [9] reported that *P. namadicus* preferred an intermediate to dry habitat characterized by highly enriched *δ*^18^O values, consistent with its C_4_-dominant grazing diet. The values observed for the Taiwan specimens, which also exhibit a C_4_-dominant diet, align more closely with *P. antiquus* and other species within the genus *Elephas*. The mean *δ*^18^O values for the subadult (−7.78‰) and adult (−6.33‰) *Palaeoloxodon* from Taiwan fall within the range observed in specimens of *E. planifrons*, *E. hysudricus* and *E. maximus,* which inhabited intermediate to wet open environments [9,41]. Our findings highlight that *Palaeoloxodon* individuals from Taiwan were feeding primarily on C_4_ vegetation (generally associated with arid environments), with oxygen isotope signals indicating access to significant water resources. We interpret that *Palaeoloxodon* from Taiwan relied on a consistent water source, probably derived from a monsoon-fed river system draining through the region, which explains the depleted *δ*^18^O values [104,105]. Moreover, given that the range of *δ*^18^O values for a river system depends on multiple factors, such as temperature, precipitation patterns, evapotranspiration, groundwater runoff, altitude, location and position within the river, these factors could further explain the observed variations of *δ*^18^O values in our specimens [106,107].

**Figure 6.**
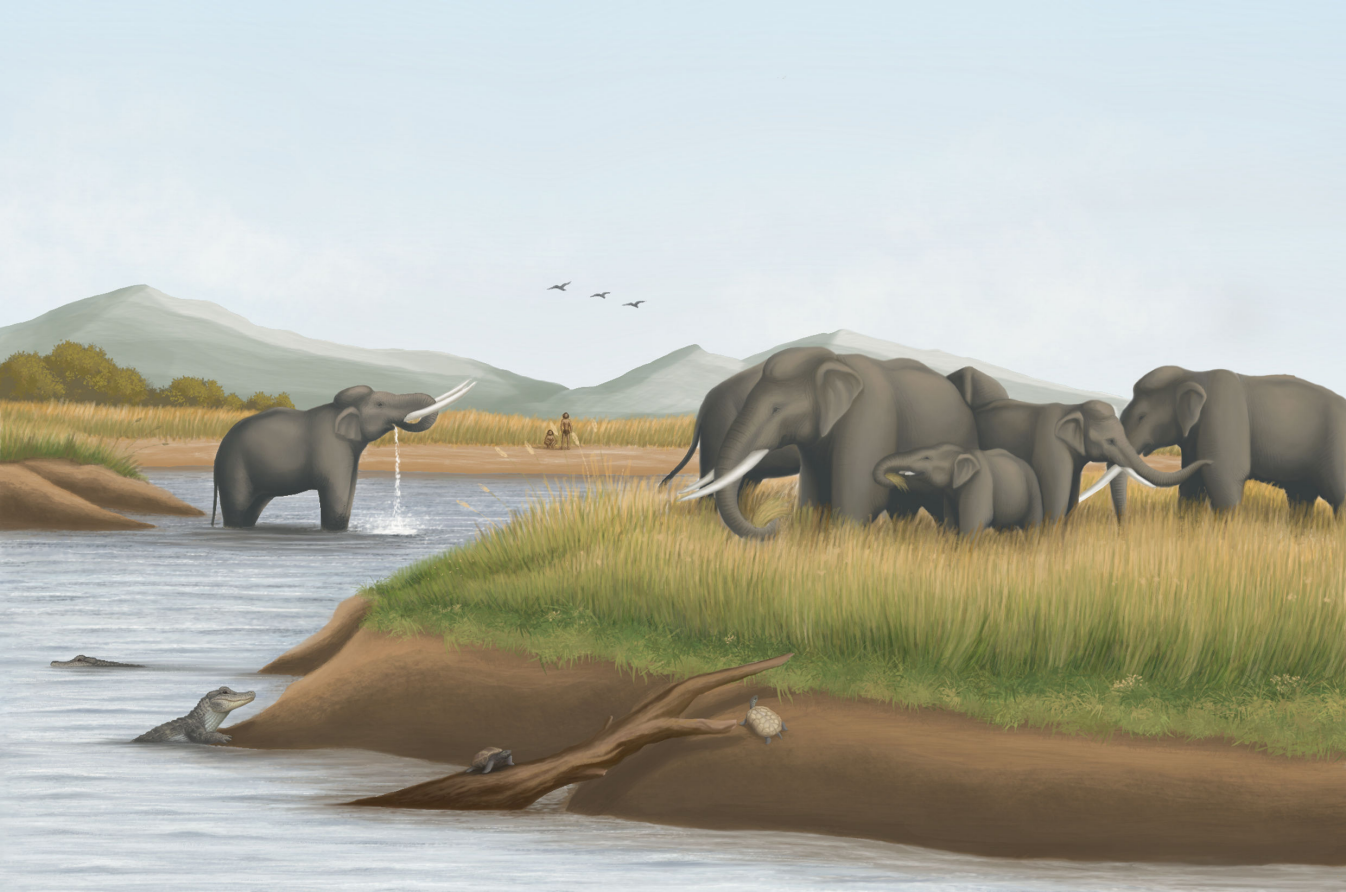
Artistic reconstruction of the Pleistocene ecosystem in the Taiwan Strait, emphasising the population of *Palaeoloxodon* in the C_4_ grasslands. Illustrated by D.S.B.

The Taiwan continental shelf, referred to as the Taiwan Strait, has a current average depth of approximately 60–70 m, with numerous underwater troughs and ridges reaching depths of about 100 m, as depicted by bathymetric data (figure 5). This shelf would have been exposed during glacial periods when global sea levels dropped. Boggs *et al.* [108] hypothesised the presence of a palaeo-river system, an extension of the Minjiang river from mainland China, during the Last Glacial Maximum when sea-levels were approximately 140 m below the present levels (see also [109]). The preserved underwater troughs and ridges are probably relics of these palaeo-drainage systems that existed during the Pleistocene. The *δ*^18^O results we obtained for the *Palaeoloxodon* specimens from the Taiwan Strait can therefore be better interpreted as evidence for the presence of such river systems during the periods of glacial maximum. Thus, the habitat for these proboscideans would have included vast C_4_ grasslands accompanied by riverine areas that served as a steady source of water (figure 6). Further research on the associated fossil taxa and deep-sea sedimentary records from the Taiwan Strait would help improve our understanding of these palaeo-hydrological systems.

## 5. Conclusion

The results of this study provide a novel insight into a vanished ecosystem that helped *Palaeoloxodon* to thrive in the Taiwan strait region during the Pleistocene. The *δ*^13^C_enamel_ of all the studied *Palaeoloxodon* specimens showed extensive reliance on C_4_ vegetation as their primary diet, having a similar grazing niche like *P. namadicus*. Furthermore, the studied specimens representing different age groups showed considerable ontogenetic variations in isotopic signatures. We identified patterns indicative of weaning behaviour in the juvenile specimen, a first for the genus *Palaeoloxodon*. The subadult specimen exhibited higher variability in both *δ*^13^C and *δ*^18^O values, indicating higher movement and land use patterns similar to extant elephants. Additionally, the *δ*^18^O results obtained for the studied specimens support the hypothesis of a palaeo-river system in the region, indicating the presence of monsoon fed-river systems during the Pleistocene glaciation periods. Our study helps reconstruct a unique and diverse Pleistocene ecosystem, characterized by C_4_ grasslands and riverine habitats, that harboured a rich extinct biodiversity which included megaherbivore clades like *Palaeoloxodon*. It contributes to the understanding that *Palaeoloxodon* was a highly adaptive group of crown elephantids that thrived in C_4_-dominated habitats in the tropics and subtropics, with multiple lineages from different geological ages exploiting these rich resources. Moreover, this research increases our understanding of the life history and biology of these colossal proboscideans. Finally, our results highlight the presence of an ecosystem distinct from present-day Taiwan, underscoring the importance of studying these vanished ecosystems and the biodiversity turnover that occurred following the extinction of these megaherbivores.

## Data Availability

Raw data and supplementary information: supplementary figures, statistical tables and compiled published data are available as part of supplementary material [110]; 3D files: https://doi.org/10.5281/zenodo.17365476. Electronic supplementary material is available online [111].
